# Heritability of Asymmetry and Lateral Plate Number in the Threespine Stickleback

**DOI:** 10.1371/journal.pone.0039843

**Published:** 2012-07-06

**Authors:** John Loehr, Tuomas Leinonen, Gabor Herczeg, Robert B. O’Hara, Juha Merilä

**Affiliations:** 1 Ecological Genetic Research Unit, Department of Biosciences, University of Helsinki, Helsinki, Finland; 2 University of Helsinki, Lammi Biological Station, Lammi, Finland; 3 Department of Mathematics and Statistics, University of Helsinki, Helsinki, Finland; University of Uppsala, Sweden

## Abstract

The estimation of individual fitness and quality are important elements of evolutionary ecological research. Over the past six decades, there has been great interest in using fluctuating asymmetry (FA) to represent individual quality, yet, serious technical problems have hampered efforts to estimate the heritability of FA, which, in turn, has limited progress in the investigation of FA from an evolutionary perspective. Here we estimate the heritability of number of lateral plates, their FA and directional asymmetry (DA) in threespine stickleback, *Gasterosteus aculeatus*. By (i) using a meristic trait and (ii) basing our calculations on a large half-sib design experiment involving 2,079 offspring from 84 families, we overcame many of the difficulties faced by earlier FA studies. Both lateral plate number and FA in lateral plates were heritable (h^2^ = 0.46 and 0.21, respectively), even after controlling for marker genotypes linked to *EDA* (the major locus influencing plate number). Likewise, DA in lateral plates was heritable h^2^ = 0.23). The additive genetic component of FA in lateral plates makes it a prime candidate for further investigation into the evolutionary implications of FA and the genetic underpinnings of developmental instability. This discovery in an evolutionary model species holds the possibility to invigorate the study of FA from an evolutionary perspective.

## Introduction

The estimation of the quality or fitness of individuals is an important part of many biological research projects, especially in evolutionary biology. One relatively easy way that has been proposed to approximate individual quality/fitness is to measure the asymmetry of bilateral characters. While directional asymmetry (a consistent bias towards a given side) and antisymmetry (consistent asymmetry towards a random side) result from normal development, fluctuating asymmetry (FA; small non directional departures from perfect symmetry) is a result of disturbed development [1], [2]. As one would expect that high quality individuals had a more stable development, they might express lower levels of FA ([3], [4], but see [5]) as the random component left after other sources of variation in asymmetry (i.e. antisymmetry or directional asymmetry) is diminished.

The study of FA was initially received with enthusiasm because it appeared to be a useful measure of individual quality in various contexts in evolutionary ecology and conservation biology research [2], [3], [6], [7]. For instance, assessment of FA can be a simple way to measure the amount of stress encountered by an individual during growth and development (greater stress can result in greater asymmetry), thus acting as a proxy measure of the underlying trait of Developmental Instability (DI) (e.g. [8]). During development individuals of higher quality may be more resistant to stress, which may be environmentally or genetically induced [9].

Despite the potential usefulness of FA, its overall value in evolutionary ecology has been questioned for a number of reasons. From an evolutionary perspective, the usefulness of FA resides in the possibility that it can predict lifetime reproductive success [10]. However, in the light of conflicting evidence, Lens *et al.* [11] cautioned that it should not be universally assumed that FA reflects fitness (also see [12], [13]). Many of the conflicting results found for FA may actually be due to difficulties in measuring it accurately [8], [14]–[16], and a lack of understanding of the underlying genetic architecture that affects development and FA [11], [13]. Even the estimation of the heritability of FA (which is inevitable for evolutionary considerations) has turned out to be a particularly ‘slippery fish’, and the struggle towards this goal has been ongoing for over half a century (reviewed in [8]).

Artificial selection experiments in *Drosophila melanogaster* have shown responses to selection indicating that FA is heritable [17]–[20]. However, the vast majority of studies, on many species, have found little or no heritability of FA. Fuller and Houle [21] found that only a small fraction of studies (15 out of 179) assessing heritability of FA found evidence for it, and possible confounding factors were present in most of those cases. Meta-analyses have also been used to combine multiple estimates of heritability of FA. While initial meta-analysis estimated the average heritability of FA to be as high as 0.27 [22] this analysis proved to be controversial, and subsequent analyses suggested much lower estimates ranging from 0.026 to 0.08 [21], [23]. The end result of all this work is the general acceptance that FA heritabilities, if present, are very small [13].

Using simulations, Fuller and Houle [21] and van Dongen [24] explored why significant heritability estimates of FA are so infrequent in the literature. These treatments point to the many pitfalls that have been unearthed over decades of research in the measurement of the heritability of FA (also see [8]). The experimental designs have frequently not been appropriate and sample sizes and sire to offspring ratios have not been optimal for producing meaningful estimates of heritability [21], [24]. Measurement error appears to be particularly difficult to overcome and can obscure results (e.g., [8], [14]). The conclusion from these studies was that the inability to find significant heritabilities for FA traces down to methodological difficulties.

The inability to determine whether there is a genetic basis for FA is unfortunate in that it has prevented progress on understanding the underlying evolutionary processes that affect development and asymmetry. In their review, Leamy and Klingenberg [13] pointed out that ”… *a better understanding of the genetic architecture of FA should provide a much-needed perspective for sorting out the sometimes unexpected or contradictory patterns of differences in FA*.” In the present manuscript our goal was to attempt to lay a foundation for future work on the investigation of the underlying genetic architecture of FA through the rigorous testing of the heritability of FA in a model trait of a model species.

An ideal trait to study the genetics and evolutionary implications of FA is the number of lateral plates of threespine sticklebacks (*Gasterosteus aculeatus*). Apart from being easy to count with little measurement error (see below), lateral plates are important structures in the defense against predatory attacks [25]. Lateral plate FA may be influenced by predators [26]–[28], and plate asymmetry is also an indicator of immunocompetence, with more asymmetric fish having higher rates of infection by endoparasites [29].

While the selective milieu influencing FA patterns in sticklebacks is reasonably well understood, results from research in heritability of lateral plate FA in threespine sticklebacks have produced conflicting estimates. Both Hagen [30] and Hermida *et al.* [31] estimated FA heritability from parent-offspring regressions arriving at estimates of 0.63 (SE  = 0.16) and −0.12 (SE  = 0.14), respectively. These estimates remain difficult to interpret because 1) the experimental designs could not properly separate environmental and genetic factors [21] and 2) the method used (total count of plates on left and right sides) to assess FA may not accurately reflect true FA ([26], and see Materials and Methods for a discussion of total count vs. homologous pairs assessment).

Here, our main aim was to estimate the heritability of FA for threespine stickleback lateral plates. We did this by conducting a large scale breeding experiment involving 42 sires and 84 dams, with a total number of 2,079 offspring, and subjecting the data to a rigorous Bayesian analysis. Hence, we were able to circumvent the two main problems faced by most previous analyses of heritability of FA: the small sample size and the large measurement error in the trait. The secondary aims of our research were to estimate the heritability of plate number and DA. The fish in our crosses varied greatly in plate number, and ranged from 30+ plates to a minimum of nine plates on one side of the fish, which correspond to full and partial plated morphs [32]. Here, we estimated the heritability of plate number and DA in sticklebacks with an approach that is statistically (cf. fitting an improved model) and genetically sound (cf. large sample size and appropriate design). Moreover, by accounting for the *EDA* (ectodysplasin A) gene in our analyses, we were able to address the question to what extent plate number is heritable after the variance due to this major gene influencing plate number [33], [34] has been accounted for.

## Results

**Figure 1:**
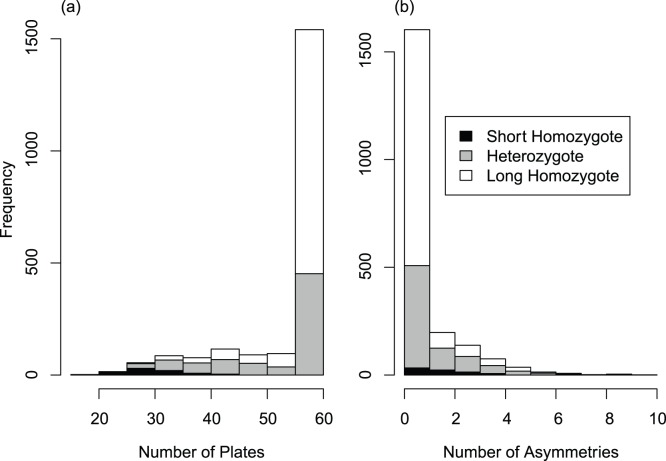
Histograms of (a) distribution of total plates on first 30 myomeres on each fish, (b) number of asymmetric myomeres, i.e. with only one plate. Shaded areas within bars show the proportion of *EDA* marker genotypes (see Table 1, Stn380) observed in the data.

**Figure 2:**
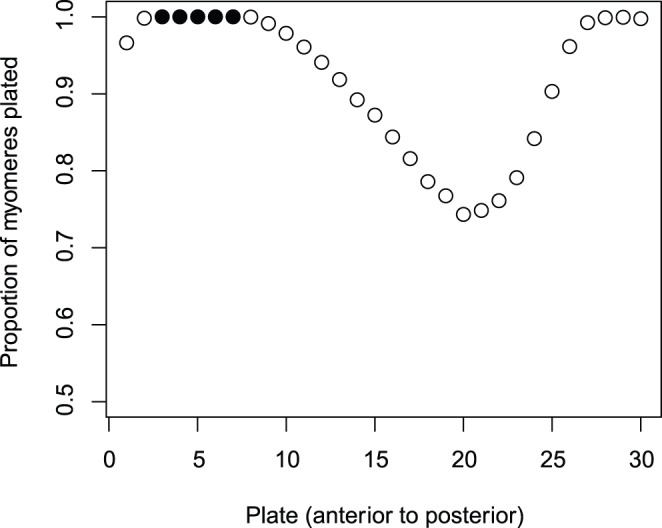
Proportion of myomeres (segments of fish) that have plates present on them in 2079 threespine sticklebacks. Filled circles: myomeres that are completely plated in all fish.

Most fish (63%) had plates at all of the myomeres, and the variation in fish that had some plates missing appeared continuous (Fig. 1a). About 35% (732 of 2,079) of all fish were asymmetric, with most only having one or two asymmetries (Fig. 1b). When plates were missing they were frequently absent between myomeres 12 and 25 (Fig. 2).

**Figure 3:**
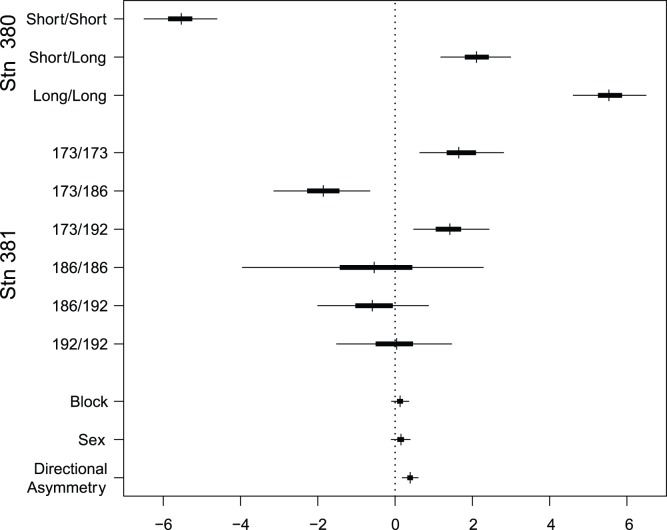
Log odds ratios of plate presence for fixed effects in the estimation plate number, directional asymmetry (DA) and fluctuating asymmetry (FA). A value of 0 means there is no effect of the factor. In ‘Sex’ refers to the effect of gender and ‘Block’ refers to block in the experimental set-up on plate number. The allelic effects associated with loci Stn 380 and Stn 381, which are closely linked to the EDA gene, also affect plate number. Posterior mode, and 50% (thick bar) and 95% (thin bar) highest posterior density intervals are shown.

Plates were directionally asymmetric with more plates on the right than left side (Fig. 3). The effects associated with the marker genotypes in the model were different (Fig. 3). Stn380 showed a large additive effect (*a* = 5.5, 95% Highest Posterior Density Interval (HPDI): 4.6–6.5): if a Long homozygote myomere has a probability of 0.99 of being plated, substituting genes to a Short homozygote changes the probability of being plated to 0.28; 93% of the genetic variance in Stn380 being additive. In contrast, only 19% of the genetic variance in Stn381 was additive: most of the variance was dominance variance, due to the contrast between genotypes including the 173 allele: the combination with allele 186 gave less plates than either the homozygote or the 173/192 genotypes (Fig. 3).

**Figure 4:**
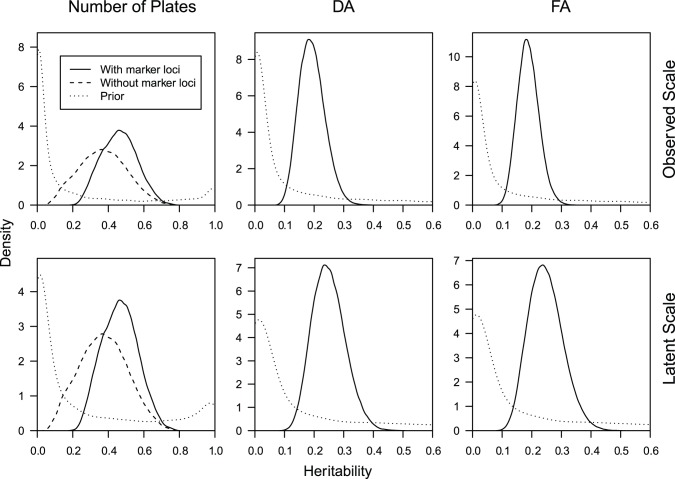
Posterior estimates of heritability for plate number, DA and FA in *G. aculeatus* lateral plates. Solid black: model including *EDA*-linked loci, dashed line: model excluding *EDA*-linked loci, dotted line: prior probability.

**Figure 5:**
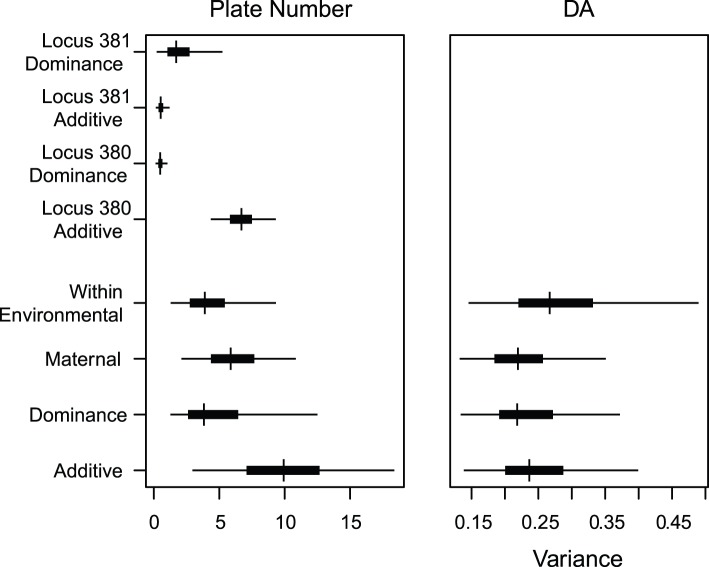
Posterior estimates of variance components for plate number, DA and FA in *G. aculeatus* lateral plates. Posterior mode, and 50% (thick bar) and 95% (thin bar) highest posterior density intervals are shown. Note that the maternal and dominance effects are partially confounded.

The heritability of lateral plate FA on the latent scale was estimated as *h^2^* = 0.24 (95% HPDI: 0.14–0.36) (Fig. 4). Similarly, the heritability of lateral plate DA on the latent scale was estimated as *h^2^* = 0.23 (95% HPDI: 0.15–0.36; Fig. 4). The heritability of plate number (also latent scale) was estimated as 0.46 (95% HPDI: 0.27–0.65) (Fig. 4). In this model, the variance explained by the two marker loci linked to *EDA* was *h^2^* = 0.19 (95% HPDI: 0.14–0.25). When the heritability of plate number is estimated without taking *EDA-*linked loci into account a somewhat lower estimate is produced 0.36 (95% HPDI: 0.12–0.62) (Fig. 4). The above heritability estimates were barely affected by the choice of model (latent or observed; Fig. 4), with the largest change being a reduction in the FA heritability to 0.18 (95% HPDI: 0.12–0.26). The variance components underlying all estimated effects for plate number, DA and FA are shown in Fig. 5.

## Discussion

The heritability estimate of *h^2^* = 0.24 for lateral plate FA is high compared to general estimates of FA heritability stemming from meta-analyses [8], [21]. To our knowledge, only one previous study using a half-sib design has found evidence for a significant heritability of FA [35]. This massive effort involved over 10,000 *D. melanogaster* individuals and estimated *h^2^* bristle count FA to be <0.05. When our results are viewed in light of previous work showing that predation pressure selects for symmetric fish [27], [36], it is evident that selection on lateral plate FA can have evolutionary consequences in populations subject to predation.

The detection of FA heritability over the last half century of research has been surprisingly difficult, yet it appears that we are now just beginning to understand what factors must be taken into account to estimate heritability of FA effectively. In this light, we examine the characteristics of our study that have aided in the detection of FA heritability. The sample size used in our study is large when compared to most other attempts to measure FA heritability and is only exceeded by work with fruit flies [35]. While our dam to sire ratio (2∶1) is well below the recommended 25 to 1 [21], the sire to offspring ratio of 1-2 to 100 advised by van Dongen [24] was fulfilled.

**Figure 6:**
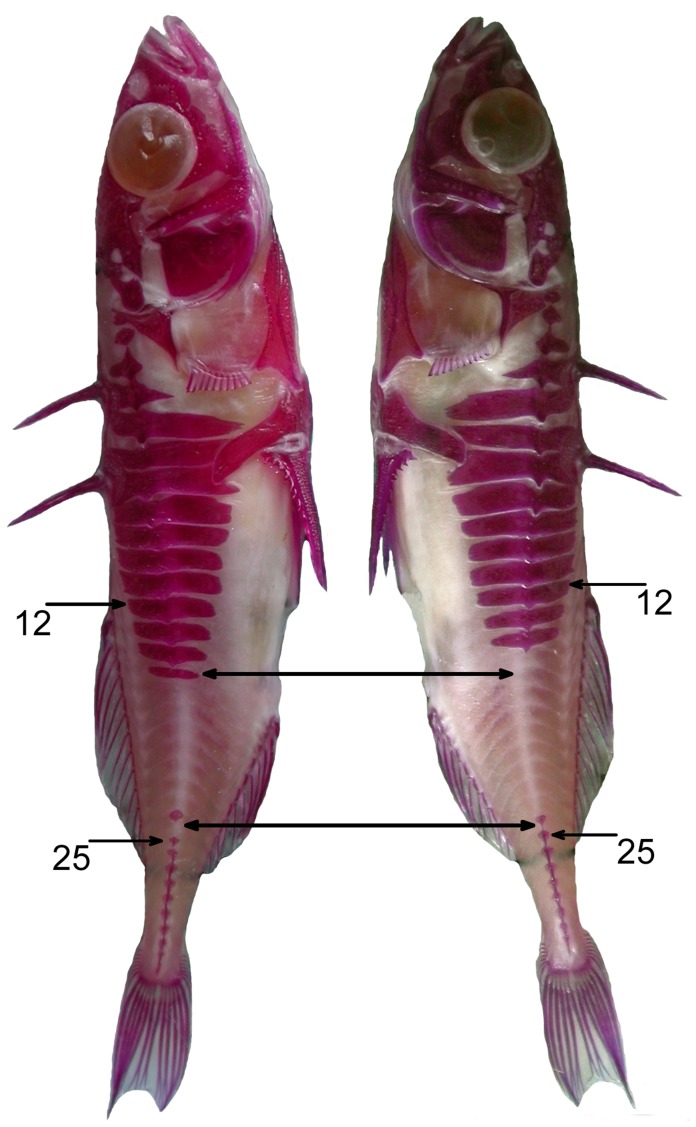
Example of a partially plated threespine stickleback stained for purposes of assessing plate morphology. Asymmetry in plates was assessed by comparing presence and absence of plates in homologous myomeres on the left and right sides of the fish (asymmetrical myomeres indicated by double headed arrows). Most variation in plate presence/absence was between the 12^th^ and 25^th^ myomeres as indicated in the figure. (Note: Background digitally removed.).

The use of a meristic trait, such as presence of lateral plates, to measure FA has some distinct advantages that may aid the accuracy to which heritability can be estimated. Meristic traits are less prone to measurement error than metric traits, because they can simply be counted as present or absent. Meristic traits also allow for a highly accurate assessment of asymmetry if the trait in question can be compared to its homologous pair on the other side of the organism (Fig. 6; [1], [36], [37]).

When FA is estimated using homologous pairs of a meristic trait, a few other important advantages emerge. Repeated measurements of the same trait can be made (although not all plates are informative for FA, see Fig. 2), and more power can be gained by more accurately modeling the probability of being plated. In our particular case, individuals with all plates will not show FA. Basing FA estimates on the total numbers of plates underestimates any underlying FA because individuals with all plates appear in the data as having zero FA. In contrast, our analysis allows them to have an underlying FA in the propensity to have plates, but this will be masked when individuals have all or nearly all plates, i.e. there is little variation in platedness to be used to estimate heritability. The information about FA thus largely comes from the partially plated individuals.

Several heritabilities can be defined, depending on how exactly the trait is defined. In practice, there was little difference between following Swain [38] and defining the trait as the liability, or as the observed FA. This suggests that the “sampling” process only has a small effect on the variance in FA, which in turn suggests that it is a good indicator of the individual's DI because the actual FA and expected FA are similar [8]. This is, in part, because we averaged over all 30 myomeres: the FA for a single binary trait will have a larger contribution from sampling variance, and may therefore provide a less accurate measure of an individual's FA.

We found a right-side bias in plate number indicating that directional asymmetry (DA) prevails in our study population and is also heritable (*h^2^* = 0.23). Although it is commonly accepted that DA is genetically determined [39], and QTL’s for DA have been found [40], additive genetic variance in DA has been shown to be lacking in a number of selection experiments [41], [42], [43]. However, two recent selection experiments have shown that DA in a population can respond to selection with changes occurring in the population mean [44], [45]. Thus, our result provides further evidence that DA can have a heritable component and has the possibility to respond to selection.

Reimchen and Nosil [29] measured asymmetry of over 10,000 adult *G. aculeatus* from a lake in western Canada. In their population there was a left side bias making the trend opposite to that found in the present study. DA in stickleback lateral plates could be due to interactions with predators or to basic biases in vertebrate developmental pathways [28]. Reimchen and Bergstrom [28] suggested that lateral plate DA may be a result of left/right biases in capture techniques of avian predators. If this is correct, our study would suggest that major predators of European populations use different capture techniques, or that the escape movement of fish is biased in opposite directions in Europe and North America.

We found that heritability of plate number was 0.46 and the variance explained by the two marker loci linked to the *EDA* gene was 0.19. This result contrasts with previous studies which have shown that the *EDA* gene can have a much stronger effect on plate number (e.g., [34], [46], [47]). In cases where *EDA* has been shown to have explained 80% of variation in plate number, other (unknown) modifier genes have been inferred to have a smaller effect [33], [34]. The difference between our results and previous ones may be found in the pattern of plate morphs in study population. In our crosses, there was large variation in plate number, which varied between 9 and 35 on one side of a fish. Although fish in our crosses occasionally had very few plates, a few posterior plates were always present. When *EDA* has shown a large effect, the populations studied have had individuals with all plates present as well as fish where all posterior plates were missing [33], [34].

An important caveat to consider is the possibility that the markers we used are not directly linked to *EDA* in our study population. However, this seems unlikely since previous research has shown that the same *EDA* alleles found by Colosimo *et al.* [33], [34] for North American populations predict plate morph in European populations [46]. Yet, as the results stand, they suggest that there may be other genetic factors beyond *EDA* having significant additive effects on plate number in a Baltic Sea population of threespine sticklebacks.

We have shown that there is significant heritability in the FA of threespine stickleback lateral plates. By identifying a trait with significant FA heritability in a model species, our work should open avenues for more detailed evolutionary and genetic investigations in the role and indicator value of FA. For instance, future work on threespine sticklebacks can begin to identify the underlying genetic architecture of FA, as well as to elucidate the implications of spatially varying predation mediated natural selection on plate FA among different populations. Furthermore, the fact that FA has a genetic basis also has implications for the use of FA in conservation biology as a measure of stress [6], [7]. While previous work has suggested that FA is purely influenced by the environment [21], [23], our results suggest that genetic *vs* environmental causes of FA should be evaluated on a case by case basis, and our results add weight to the idea that the inability to find significant heritabilities for FA may be due to methodological difficulties inherent to the study of FA [21], [24].

## Materials and Methods

### Ethic Statement

The experiment was approved and conducted under the license (HY 121-06) from the Helsinki University Animal Experimentation Committee.

### Sampling and Fish Rearing

Mature male and gravid female threespine sticklebacks were collected during the breeding season in June 2006 from a Baltic Sea population (Vuosaari, Helsinki; 60°10′N, 25°00′E). A seine net with 6 mm mesh size was used for trapping. The fish were transported to the laboratory in 30 L tanks supplied with a battery-operated air pump, and the crosses were made immediately upon arrival. The crosses were performed using a nested paternal half-sib design, i.e. each male was crossed with two different females (North Carolina I design). In total, 42 males were crossed with 84 females. Males were anaesthetized and killed with an overdose of MS-222 (tricane methanesulphonate) before the testes were extracted and finely chopped in a few drops of water. The sperm solution was used to fertilise eggs *in vitro*, which were obtained by gently pressing the abdomen of the ripe females. The fertilised eggs were placed in cylindrical plastic containers with a plastic mesh bottom. The containers were submerged in 10 L plastic tanks with air supply to keep the water saturated with oxygen. Throughout the experiment the water temperature was set to 17 °C and the photoperiod to 12 L : 12 D. Once the eggs hatched, each clutch was divided into two replicate 10 L plastic tanks, and fed daily to excess with *Artemia* sp. nauplii. After three months, the fish were also fed daily with chopped chironomid larvae.

Initially 25 sticklebacks were kept in each replicate tank, two months after hatching, to ensure the fish did not suffer stress from abnormally high density, the number of fish was reduced to 15. To accomplish this, all fish were caught in a hand net and then 15 fish were randomly picked, so as to avoid any potential bias in catching fish. Families with less than 15 fish per replicate (11 out of 84 half sib families) were pooled with other families to make the density in each tank 15. The fish from different families were marked with fluorescent elastomers (Northwest Marine Technology, Inc.) prior to pooling. The fish were killed after six months, when they had reached ca. 4 cm in standard length and thus completed their lateral plate development [32], [48]. The fish were fixed in 10% formalin and stored horizontally for a minimum of one month, and then stained with Alizarin Red S using the procedure described by Pritchard and Schluter [49].

### Lateral Plate Asymmetry

The presence or absence of each lateral plate on both sides of each myomere for 2,079 fish was assessed from photographs (Fig. 6). We modeled presence/absence of plates at homologous myomeres on both sides of the fish [26], [36]. This leads to a more accurate measure of asymmetry than comparing total counts of plates on both sides, as it avoids asymmetries canceling out. However, error can occur if myomere location or plate presence/absence is incorrectly assessed. For the analyses, we only included the 30 first anterior myomeres (following Bergstrom and Reimchen [26]), as the small size of the posterior keel plates and myomeres can reduce accuracy of presence/absence observations. Bergstrom and Reimchen [26] assessed the amount of error versus the amount of asymmetries in their study and found that error was about five times less than the incidence of asymmetry. Following their example, we took a random sample of 40 individuals and reassessed plate presence/absence. We found that error occurred in 0.67% of myomeres, while asymmetries were found in 3.42% of myomere positions. Thus, error was about five times less than the incidence of asymmetry also in our study, suggesting that the results accurately reflect FA.

### Sex Identification and *EDA* Genotyping

Individuals were sexed using DNA obtained from fins. DNA was extracted following Duan and Fuerst [50], and sex identification was done by amplifying a part of 3′UTR of Idh-gene [51]. The final PCR reaction volume was 10 µl and consisted of 1 µl template DNA diluted 1∶10, 1x NH4 Reaction Buffer (Bioline), 1.5 mM MgCl_2_ (Bioline), 20 µM of each dNTP (Finnzymes), 0.32 µM of Idh exon II 37F and Idh exon II 290R primers, as well as 0.25 U of BioTaq polymerase (Bioline). PCRs were conducted with an MBS thermal cycler (Thermo) according to following thermal profile: 94°C for 3 min, 38 cycles of 94°C for 30 s, 56°C for 30 s, 72°C for 1 min and the final extension at 72°C for 5 min. After PCRs, 5 µl of amplicon was run on a 2% Agarose LE (Cambrex) with 1x Loading dye (Fermentas). Fragment sizes were determined against a size standard (GeneRuler, Fermentas). Males had two fragments (∼280 bp and 300 bp), females only one (300 bp). Each PCR plate contained certified male DNA as a positive control and a negative control (no sample). In some cases - either due to failed DNA-extraction or poor quality of DNA - the sex-specific region failed to amplify despite the further optimization of used PCR-protocol.

To investigate the effect of a known genetic determiner (*EDA* gene, [33], [34]) on plate number and FA, individuals were typed for two *EDA*–linked microsatellite loci using primers for Stn380 and Stn381 [34]. PCRs were conducted in total reaction volume of 10 µl with 1xMultiplex Mastermix, 1 µl Q-solution (206145, Qiagen) and 5 pmol of each primer (forward primers labeled with fluorochromes FAM and TET) together with 1 µl of template. Temperature profile consisted of preliminary denaturation at 95°C for 15 min followed by 34 cycles in 95°C for 30 s, 56°C for 90 s, 72°C for 1 min and final extension at 60°C for 5 min. Amplicons were run diluted 1∶100 together with ET-ROX 400 size standard in MegaBACE1000 (GE Healthcare) capillary electrophoresis instrument and scored in Fragment Profiler 1.2 software (GE Healthcare).

To avoid possible run-to-run and capillary-to-capillary allele size differences together with reader based errors, the Flexibin algorithm [52] was used to estimate the final allele sizes. For each lot of 94 samples, an individual from already published dataset [53] as well as a negative (no template) control were run and scored. Divergent alleles were verified by re-running PCR and capillary electrophoresis.

### Statistical Analyses

#### Plate Model

Presence of a plate on a myomere (on one side of a fish) was modeled as a Bernoulli trial, so that.
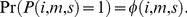
(1)for individual *i* the plate on side *s* of myomere *m*. This was modeled as a logistic regression:
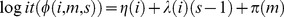
(2)which separates the probability into individual-level effects of the number of plates (*η*(*i*)), the asymmetry (*λ*(*i*)), and a plate effect (*π*(*m*)): *sE*{1,2}, so the asymmetry effect is for *s* = 2, or the right hand side of the fish. *η*(*i*) and *λ*(*i*) are both constant over myomeres, so we can interpret the model as a repeated measures model over the myomeres of the fish.

#### Individual Level Model

We model three individual-level traits related to platedness: the number of plates, directional asymmetry (DA - i.e. the overall mean asymmetry in platedness) and fluctuating asymmetry (FA - i.e. random asymmetry fluctuating around DA). For each of these we use a standard genetic model [54] to model the trait, *µ_t_*(*i*) (*t* = 1,2,3 for number of plates, DA and FA respectively), at the individual level:
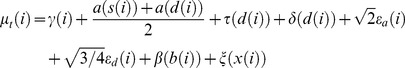
(3)where

- α(*s*(*i*)) and *α*(*d*(*i*)) are the parents' breeding values
- *τ*(*d*(*i*)) and *δ*(*d*(*i*)) are the maternal and dominance effects: these are partially confounded because of the experimental design, so are not interpreted further.
- *ε*_a_(i) and *ε*_d_(*i*) are the additive and dominance deviations, due to segregation.
- *ε*_e_(*i*) is the residual/within-individual environmental deviation
- *β*(*b*(*i*)) is the block effect (each block was one 10 liter tank), for an individual *i* grown in block *b*(*i*). This is constrained
- *ξ*(*x*(*i*)) is the sex effect. This is constrained so that the sum of the effects is zero.

*β*(*b*(*i*)) and *ξ*(*x*(*i*)) were each constrained so that so that the sum of their effects were zero. The other parameters are modeled as random effects, with variances given by the standard quantitative genetic model for the additive (*V_Add_*), maternal (*V_Mat_*), dominance (*V_Dom_*) and within-family environment (*V_EnvW_*) respectively:
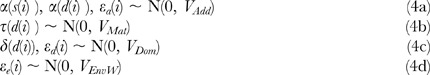



#### Plate effect and EDA

The plate effect is.
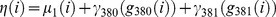
(5)where *γ*_380_() and *γ*_381_() were the effects of the *EDA* locus, was modeled through the joint effects of the two genetic markers, with genotypes *g*_380_(i) and *g*_381_(i).

Stn380 had five alleles, of which three were rare (Table 1), so this was simplified to two genotypes: Long (>190 bps) and Short (<190 bps). We thus used the standard bi-allelic model (e.g. [54] chapter 4) with the homozygotes (*γ*_380_(1) and *γ*_380_(3)) having effects −*a* and +*a* (for Short and Long respectively), and the heterozygote (*γ*_380_(2)) having an effect *d*. The additive genetic variance due to this locus was then calculated as *V_Add_*(380)  = 2*p*_380_(1−*p*_380_)(*a*+(1−2*p*_380_)*d*)^2^, and the dominance variance is *V_Dom_*(380)  = 4*p*_380_^2^(1−*p*_380_)^2^(*a*+(1−2*p*_380_)*d*)^2^, where *p*_380_ is the frequency of the Long allele.

**Table 1:** Allele frequencies in microsatellite loci Stn380 and Stn381 which are strongly linked to the *EDA* gene [34]. For analysis the alleles of Stn380 were simplified to two genotypes: Long (>190 bps) and Short (<190 bps).

Stn380		
Allele	Absolute Frequency	Relative Frequency (%)
181	14	0.4
183	5	0.1
185	2991	77.0
197	798	20.5
199	78	2.0
**Stn381**		
**Allele**	**Absolute Frequency**	**Relative Frequency (%)**
173	2798	72.2
186	95	2.5
192	983	25.4

The Stn381 locus had three alleles (Table 1), and all three were used in the estimation of the genotypic effect. A separate effect was estimated for each genotype, so six genotypic effects (*γ*_381_(*j*) for *j* = 1,…,6) were estimated in total, each with frequency *p*_381_(*i*). The genotypic effects were assumed to be normally distributed, i.e. *γ*_381_(*i*) ∼ *N*(0, *V*_381_). From this the average deviation of each genotype from the mean was calculated, and hence the breeding value, as the average effect of that gene in its offspring. The additive variance is then the weighted (by the frequency of the genotype) mean squared breeding value. The dominance effect for each genotype was calculated as the difference between the genotypic value and the breeding value of the genotype, and the dominance variance then calculated as the weighted average squared dominance effect (see Appendix for details). The additive genetic variance due to Stn381, *V_Add_*(381), is thus the mean squared breeding value, and the dominance genetic variance, *V_Dom_*(381), is the mean squared deviation from the breeding value [54].

#### Asymmetry

The model above defines the asymmetry effect as zero for the “left” side of the fish, and was estimated for the “right” side. Both a directional effect and fluctuating effect were modeled: the direction of the fluctuating effect had to be allowed to fluctuate between observations in an individual. The asymmetry effect was thus:
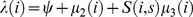
(6)where *ψ* is the mean directional asymmetry, *μ*_2_(*i*) and *μ*_3_(*i*) are the DA and FA terms (see above), and *S*(*i*,*s*) denotes the random direction of the FA effect: it can take values -1 or 1, with probability 0.5.

### Model Fitting

The fixed effect sex had to be modeled because 12% of the individuals could not be sexed. It was assumed to be a Bernoulli random variable with probability *p_s_*. Similarly, 6.6% and 6.8% of the Stn380 and Stn381 genotype data, respectively. were missing. These missing values were imputed assuming they had been drawn from a Dirichlet distribution [55].

The model was fitted with a Bayesian approach. The priors were chosen to be vague. For the variance components, wrapped t-distribution priors were placed on the variances [56]:
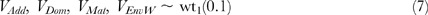
where wt*_ν_*(*σ*) is a wrapped t distribution (i.e it is restricted to values >0) with *ν* “degrees of freedom” and scale *σ*. This is only weakly informative for the variance components in the range of likely values. The other priors were:
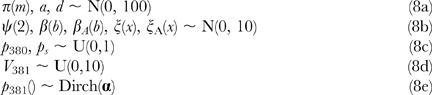



(Note the constraint that the *β*(*b*)′s, *β*_A_(*b*)′s, *ξ*(*x*)′s and ξ_A_(*x*)′s each sum to zero). Where Dirch(**α**) is a Dirichlet distribution with **α** being a vector of the same length as *p*_381_: here length 6, and we set every element of **α** to 1.

The model was fitted in OpenBUGS. 4 chains were run. After a burn-in of 10,000 iterations, another 10^6^ iterations were run, giving 4×10^6^ draws from the posterior. Convergence was judged by eye. Most parameters mixed well, with the exception of *π*(), a, *d*, and g_381_(). But even these poorly mixing variables had effective samples size [57] of at least 200.

### Heritability

Depending on our exact focus, we can calculate heritability in different ways. Swain (1987) recommended that for models of meristic traits (such as plate number) our attention should focus on the liability level, i.e. the trait of interest is the 'propensity' to be plated (here this is the log odds of any myomere being plated on one side). If we take this approach, we can calculate heritability on the liability scale:
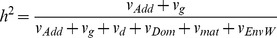
(9)where *V_g_* and *V_d_* are respectively the additive and dominance variance components due to the plate genotype, i.e. *V_g_* = *V_Add_*(380)+*V_Add_*(381) and *V_d_* = *V_Dom_*(380)+*V_Dom_*(381). The definition of heritability used above avoids the problem that the means and variance of the trait may differ [38]. However it does this by redefining the trait to remove a component of the environmental variation (i.e. the “sampling” variation, due to the trait being a realization of a random process).

We can calculate an approximate heritability for the trait on the trait scale (i.e. probability) by calculating the binomial sampling variance at the trait mean. For lateral plates, the trait is the number of plates divided by the maximum number of plates ( = 2*M* = 60). The variance of this, Var(*Pl*), is the sum of the variances in the individual plate effects. We can calculate *p*(*i*,*m,s)* from the model, evaluating it at the mean over the population. This is exp(*π*(*m*))/1+exp(*π*(*m*))) for the left hand side of the fish, and exp(*π*(*m*)+*ψ*(*s*))/1+exp(*π*(*m*)+ψ(*s*))) for the right hand side.
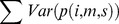
(10)


The other variance components then need to be transformed from the liability scale (i.e. the logit scale) onto the same scale. We can make this transformation (approximately) with the delta method, i.e.
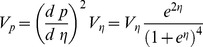
(11)where *p* is the probability at the expected value of the trait, *p* = e*^η^*/(1+e*^η^*), and *V_p_* and *V_η_* are the variances on the *p* and *η* scales.

For DA and FA, the trait of interest is the proportion of myomeres that are asymmetric. The probability that a myomere is asymmetric is *q*(*i*,*m*) = *p*(*i*,*m,1)*(1-*p(i,m,2)*)+*p(i,m,2)*(1-*p(i,m,1)*). The variance in FA is thus:
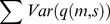
(12)


Note that we are summing over myomeres, as it is pairs of plates that are asymmetric.
