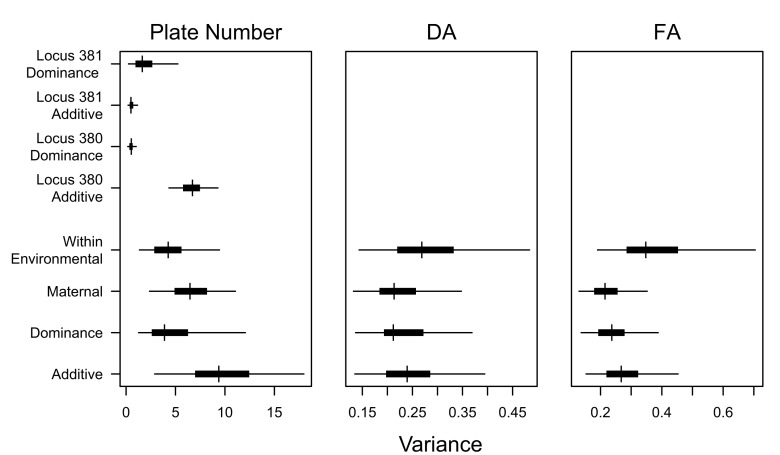# Correction: Heritability of Asymmetry and Lateral Plate Number in the Threespine Stickleback

**DOI:** 10.1371/annotation/25cec93d-c03c-46e4-b538-a9d3f584e00e

**Published:** 2013-02-25

**Authors:** John Loehr, Tuomas Leinonen, Gabor Herczeg, Robert B. O’Hara, Juha Merilä

There is an error in Figure 3. The correct Figure 3 can be seen here: 

**Figure:**
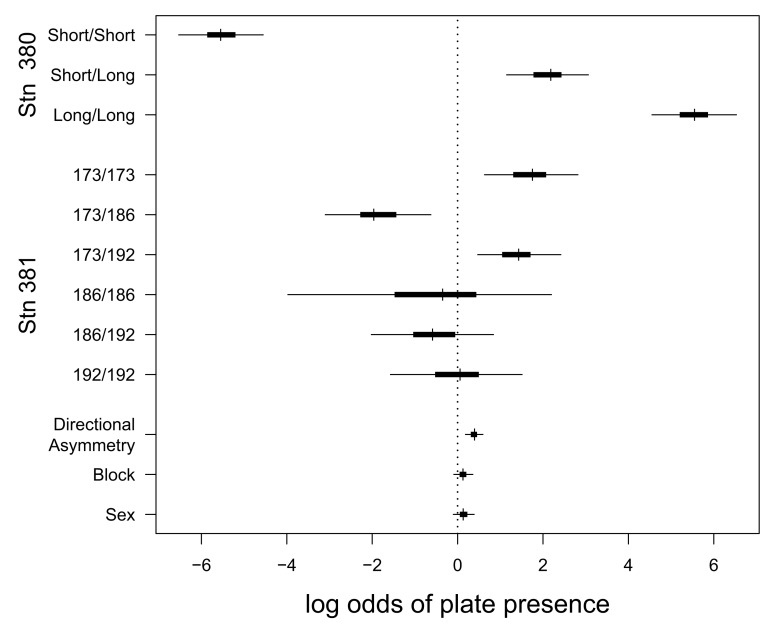


The correct legend for Figure 3 is: 

**Log odds ratios of plate presence for fixed effects in the estimation plate number, and directional asymmetry (DA).**

A value of 0 means there is no effect of the factor, a positive value means a higher probability of being plated (for DA this refers to the right side of the fish). In ‘Sex’ refers to the effect of gender and ‘Block’ refers to block in the experimental set-up on plate number. The allelic effects associated with loci Stn 380 and Stn 381, which are closely linked to the EDA gene, also affect plate number. Posterior mode, and 50% (thick bar) and 95% (thin bar) highest posterior density intervals are shown.

There is an error in Figure 5. The correct Figure 5 can be seen here: 

**Figure:**